# Supplementary material for: Correcting for sparsity and interdependence in glycomics by accounting for glycan biosynthesis
Source: Nat Commun. 2021 Aug 17;12:4988. doi: 10.1038/s41467-021-25183-5 (PMC8371009; doi:10.1038/s41467-021-25183-5)
Supplement: Supplementary file 1 — Supplementary Information [file 41467_2021_25183_MOESM1_ESM.pdf]

Supplementary information

Correcting for sparsity and interdependence in glycomics by accounting for glycan biosynthesis

Bokan Bao[1,2,3,+], Benjamin P. Kellman[1,2,3,+], Austin W.T. Chiang[2,4], Yujie Zhang[1], James T. Sorrentino[1,2,3], Austin K. York[1], Mahmoud A. Mohammad[5], Morey W. Haymond[5], Lars Bode[2], Nathan E. Lewis[1,3,4,6,*]

[1] Department of Pediatrics, University of California, San Diego, La Jolla, CA 92093, USA

[2] Bioinformatics and Systems Biology Graduate Program, University of California, San Diego, La Jolla, CA 92093, USA

[3] Department of Bioengineering, University of California, San Diego, La Jolla, CA 92093, USA

[4] The Novo Nordisk Foundation Center for Biosustainability at the University of California, San Diego, La Jolla, CA 92093, USA

[5] Department of Pediatrics, Children's Nutrition Research Center, US Department of Agriculture/Agricultural Research Service, Baylor College of Medicine, Houston, Texas 77030, USA

[6] National Biologics Facility, Technical University of Denmark

[+] These authors contributed equally to this work

**Supplementary Tables**	**3**
**Supplementary Figures**	**10**
**Supplementary Discussion**	**30**
**Supplementary References**	**31**

Supplementary Table

Supplementary Table 1 – Glossary of analyses terms

Substructures types examined in each section

Results section	Substructure	Explanation
GlyCompare decomposes glycoprofiles to facilitate glycoprofile comparison	Glyco-motif	EPO clustering was done with glyco-motif abundance
GlyCompare decomposes glycoprofiles to facilitate glycoprofile comparison	All	Overview of methods discusses every substructure type.
GlyCompare accurately clusters glycoengineered EPO samples	Glyco-motif	EPO clustering was done with glyco-motif abundance
GlyCompare summarizes structural change across glycoprofiles	Representative substructure	EPO clusters were examined for enrichment and depletion of representative substructures
GlyCompare reveals phenotype-associated substructures and trends invisible at the whole glycan level	Substructure	All HMO substructures were used to avoid merging substructure matching known HMOs. This was necessary to allow comparison to know structures
GlyCompare identifies condition-specific synthesis dynamics	Substructure	All HMO substructures were used to avoid merging substructure matching known HMOs. This was necessary to allow comparison to know structures
GlyCompare increases statistical power of glycomics data	Glyco-motif	Just HMO glyco-motifs were used to avoid artificially overpowering the analysis.

Supplementary Table 2 | HMO abbreviations

HMO abbreviations are specified in this table. Complete GlycoCT structures for all HMO and EPO glycans used in this study, can be accessed in directory ./example_data/paper_epo/glycoct/ and ./example_data/paper_hmo/glycoct/ from Zenodo database under accession code doi.org/10.5281/zenodo.5083029

HMO	Abbreviation	GlyTouCan Accession
LNT	Lacto-N-tetrose	G45827GY
LNnT	Lacto-N-neotetrose	G48059CD
2'FL	2'-fucosylactose	G10422IZ
3FL	3-fucosylactose	G06210XB
3'SL	3'-sialyllactose	G91237TK
LNFPI	Lacto-N-fucopentose I	G01650PH
LNFPII	Lacto-N-fucopentose II	G98173LG
LNFPIII	Lacto-N-fucopentose III	G83916HL
LSTb	LS-tetrasaccharide b	G19017MP
LSTc	LS-tetrasaccharide c	G72506RN
DSLNT	Disialyllactose-N-tetrose	G38710SX
DFLNT	Difucosyllacto-N-tetrose	G70115XG
FLNH	Fucosyllacto-N-hexose	G24504JY
DSLNH	Disialyllacto-N-hexaose	G47928KI
DFLNH	Difucosyllacto-N-hexaose	G63053GR
FDSLNH	Fucodisialyllacto-N-hexaose	N/A

Supplementary Table 3 | Complete information on generalized estimating equation models

The tables below specify the coefficient summary, confidence intervals, Wald test p-values. We report general model statistics including number of observations and groups, and degrees of freedom. We report effect size with marginal correlation for gaussian regressions and entropy for logistic regression. Finally, for gaussian regressions we report the Shapiro-Wilk's p-value for normality of a distribution **a.** Gaussian GEE, predicting motif abundance from secretor status while controlling for DPP **b.** Gaussian GEE, predicting motif abundance from DPP split on secretor status **c.** Logistic GEE, predicting secretor status from estimated flux while controlling for DPP

a

GEE (z(log(X62 + e)) ~ log(DPP) + Secretor ,id=subject,corstr='exchangeable')

	Coef	95% CI	Pr(Wald)	N. Obs	N. Groups	Marginal	R^2	df	Pr(Shapiro-Wilks)
(Intercept)	0.74030	(0.3485 - 1.132)	6.118e-03	47	6	0.45		44	0.53
Secretor	-1.36800	(-0.6422 - -2.095)	4.329e-07						
log(DPP)	0.09137	(0.06535 - 0.1174)	0.5294						

GEE (z(log(LSTb + e)) ~ log(DPP) + Secretor ,id=subject,corstr='exchangeable')

	Coef	95% CI	Pr(Wald)	N. Obs	N. Groups	Marginal	R^2	df	Pr(Shapiro-Wilks)
(Intercept)	1.18000	(0.5933 - 1.766)	3.288e-06	47	6	0.76		44	0.02
Secretor	-1.81000	(-0.9251 - -2.695)	3.976e-13						
log(DPP)	0.01147	(0.009961 - 0.01298)	0.8642						

GEE (z(log(DSLNT + e)) ~ log(DPP) + Secretor ,id=subject,corstr='exchangeable')

	Coef	95% CI	Pr(Wald)	N. Obs	N. Groups	Marginal	R^2	df	Pr(Shapiro-Wilks)
(Intercept)	0.7765	(0.285 - 1.268)	0.01619	47	6	0.34		44	0.22
Secretor	0.1627	(0.1216 - 0.2038)	0.2067						
log(DPP)	-0.4691	(-0.3124 - -0.6257)	5.899e-3						

GEE (z(log(DSLNH + e)) ~ log(DPP) + Secretor ,id=subject,corstr='exchangeable')

	Coef	95% CI	Pr(Wald)	N. Obs	N. Groups	Marginal	R^2	df	Pr(Shapiro-Wilks)
(Intercept)	-0.7406	(-0.5004 - -0.9808)	7.600e-06	47	6	0.36		44	0.07
Secretor	-0.2254	(-0.1115 - -0.3393)	0.3820						
log(DPP)	0.4740	(0.3884 - 0.5597)	2.682e-07						

b

GEE (z(log(X62 + e)) ~ log(DPP) + Secretor ,id=subject,corstr='exchangeable',data= 'just-secretors')

	Coef	95% CI	Pr(Wald)	N. Obs	N. Groups	Marginal R^2	df	Pr(Shapiro-Wilks)
(Intercept)	-0.742	(-0.48819 - -0.9957)	2.12e-05	31	4	0.268	29	0.605
log(DPP)	0.399	(0.33284 - 0.46529)	2.44e-06					

GEE (z(log(X62 + e)) ~ log(DPP) + Secretor ,id=subject,corstr='exchangeable',data= 'just-non-secretors')

	Coef	95% CI	Pr(Wald)	N. Obs	N. Groups	Marginal R^2	df	Pr(Shapiro-Wilks)
(Intercept)	1.218	(1.0705 - 1.3654)	$< 2e-16$	16	2	0.687	14	0.881
log(DPP)	-0.657	(-0.63058 - -0.6832)	$< 2e-16$					

GEE (z(log(LSTb + e)) ~ log(DPP) + Secretor ,id=subject,corstr='exchangeable',data= 'just-secretors')

	Coef	95% CI	Pr(Wald)	N. Obs	N. Groups	Marginal R^2	df	Pr(Shapiro-Wilks)
(Intercept)	-0.393	(-0.13891 - -0.64786)	0.233	31	4	0.0884	29	0.9928
log(DPP)	0.217	(0.17095 - 0.26372)	0.046					

GEE (z(log(LSTb + e)) ~ log(DPP) + Secretor ,id=subject,corstr='exchangeable',data= 'just-non-secretors')

	Coef	95% CI	Pr(Wald)	N. Obs	N. Groups	Marginal R^2	df	Pr(Shapiro-Wilks)
(Intercept)	0.666	(-0.11446 - 1.4473)	0.264959	16	2	0.206	14	0.928
log(DPP)	-0.359	(-0.28515 - -0.43372)	0.000653					

GEE (z(log(DSLNT + e)) ~ log(DPP) + Secretor ,id=subject,corstr='exchangeable',data= 'just-secretors')

	Coef	95% CI	Pr(Wald)	N. Obs	N. Groups	Marginal R^2	df	Pr(Shapiro-Wilks)
(Intercept)	0.743	(0.16746 - 1.3195)	0.060	31	4	0.2237	29	0.0205
log(DPP)	-0.389	(-0.17451 - -0.60313)	0.167					

GEE (z(log(DSLNT + e)) ~ log(DPP) + Secretor ,id=subject,corstr='exchangeable',data= 'just-non-secretors')

	Coef	95% CI	Pr(Wald)	N. Obs	N. Groups	Marginal R^2	df	Pr(Shapiro-Wilks)
(Intercept)	1.081	(0.90883 - 1.2538)	$< 2e-16$	16	2	0.541	14	0.222
log(DPP)	-0.583	(-0.55665 - -0.60976)	$< 2e-16$					

GEE (z(log(DSLNH + e)) ~ log(DPP) + Secretor ,id=subject,corstr='exchangeable',data= 'just-secretors')

	Coef	95% CI	Pr(Wald)	N. Obs	N. Groups	Marginal R^2	df	Pr(Shapiro-Wilks)
(Intercept)	-0.993	(-0.42991 - -1.5561)	5.99e-04	31	4	0.448	29	0.245
log(DPP)	0.528	(0.39235 - 0.66399)	5.68e-05					

GEE (z(log(DSLNH + e)) ~ log(DPP) + Secretor ,id=subject,corstr='exchangeable',data= 'just-non-secretors')

	Coef	95% CI	Pr(Wald)	N. Obs	N. Groups	Marginal R^2	df	Pr(Shapiro-Wilks)
(Intercept)	-0.662	(-0.55101 - -0.77302)	1.01e-14	16	2	0.2028	14	0.0203
log(DPP)	0.357	(0.312 - 0.4021)	2.91e-08					

c

GEE (logit(Secretor) ~ log(DPP) + X62 / X40 ,id=subject,corstr='exchangeable')

	Coef	95% CI	Pr(Wald)	N. Obs	N. Groups	Marginal Entropy	df
(Intercept)	2.001310	(-1.39521 - 5.39784)	0.4230	47	6	0.50	44
log(DPP)	0.999898	(0.999763 - 1.00003)	0.1417				
I(X62/X40)	0.989874	(0.957091 - 1.02266)	0.5469				

GEE (logit(Secretor) ~ log(DPP) + X106 / X62 ,id=subject,corstr='exchangeable')

	Coef	95% CI	Pr(Wald)	N. Obs	N. Groups	Marginal Entropy	df
(Intercept)	2.071930	(-1.48709 - 5.63095)	0.4058	47	6	0.50	44
log(DPP)	0.999989	(0.998646 - 1.00133)	0.9873				
I(X106/X62)	0.948740	(0.907258 - 0.990221)	0.0183				

Supplementary Table 4 | Table information for Supplementary Figure 11

a. Quartile information and sample size (n) for Supplementary Figure 11a.

b. Quartile information and sample size (n) of the Supplementary Figure 11b.

**a**								
IgG	truth	type	min	Q1	median	Q3	max	Sample(n)
1	F	motif	-0.5384	-0.0983	-0.0055	0.0887	1	1252
1	F	glycan	-0.4874	-0.0783	-0.0109	0.0597	1	1384
1	T	motif	0.202	0.3331	0.4048	0.5074	0.8055	192
1	T	glycan	-0.0646	0.255	0.4214	0.522	0.8079	216
2	F	motif	-0.5248	-0.0928	-0.0077	0.0759	1	1252
2	F	glycan	-0.6265	-0.0729	-0.008	0.0603	1	1384
2	T	motif	0.1666	0.3502	0.4574	0.5381	0.71	192
2	T	glycan	-0.3031	0.1798	0.3843	0.5445	0.7704	216
4	F	motif	-0.6441	-0.1928	-0.0998	0.1218	1	220
4	F	glycan	-0.6354	-0.2092	-0.0582	0.0333	1	280
4	T	motif	0.3385	0.4713	0.5495	0.6628	0.7642	104
4	T	glycan	-0.3013	0.1305	0.4522	0.5826	0.7783	120

b								
Enzyme	Truth	Type	min	Q1	median	Q3	max	Sample(n)
B4GALT	T	motif	0.1666	0.4417	0.4889	0.5958	0.8055	40
B4GALT	T	glycan	-0.1309	0.2322	0.4051	0.4771	0.7369	40
ST6GAL1	T	motif	0.202	0.3443	0.4566	0.5547	0.7642	80
ST6GAL1	T	glycan	-0.3031	0.043	0.2707	0.5355	0.7315	80

Supplementary Table 5 | Quartile information for Supplementary Figure 12.

	Tissue	Min	Q1	Median	Q3	Max
GD3	Retina	0.06	0.095	0.18	0.43	0.87
	Brain	0.02	0.05	0.09	0.47	0.91
	Plasma	0.1	0.3	0.33	0.435	0.67
GD3-Substr	Retina	0.56	0.603	0.646	0.747	0.836
	Brain	0.367	0.385	0.438	0.574	0.69
	Plasma	0.348	0.387	0.508	0.522	0.647
GM2	Retina	0	0.01	0.03	0.49	0.99
	Ciliary.Body	0.17	0.245	0.26	0.355	0.83
GM2-Substr	Retina	0.119	0.267	0.4	0.549	0.783
	Ciliary.Body	0.529	0.589	0.744	0.7778	0.904

Supplementary Figures

Supplementary Figure 1 | The substructure network of EPO dataset

The merged substructure network from 16 glycoprofiles contains 516 synthesizable glyco-substructures. The edges are colored with enzyme family, AsiaT (purple), MgatT (blue), Fut (red), B4galT (orange), iGnt (cyan), ManII (green) and the node color is according to the existence times in 16 glycoprofiles.

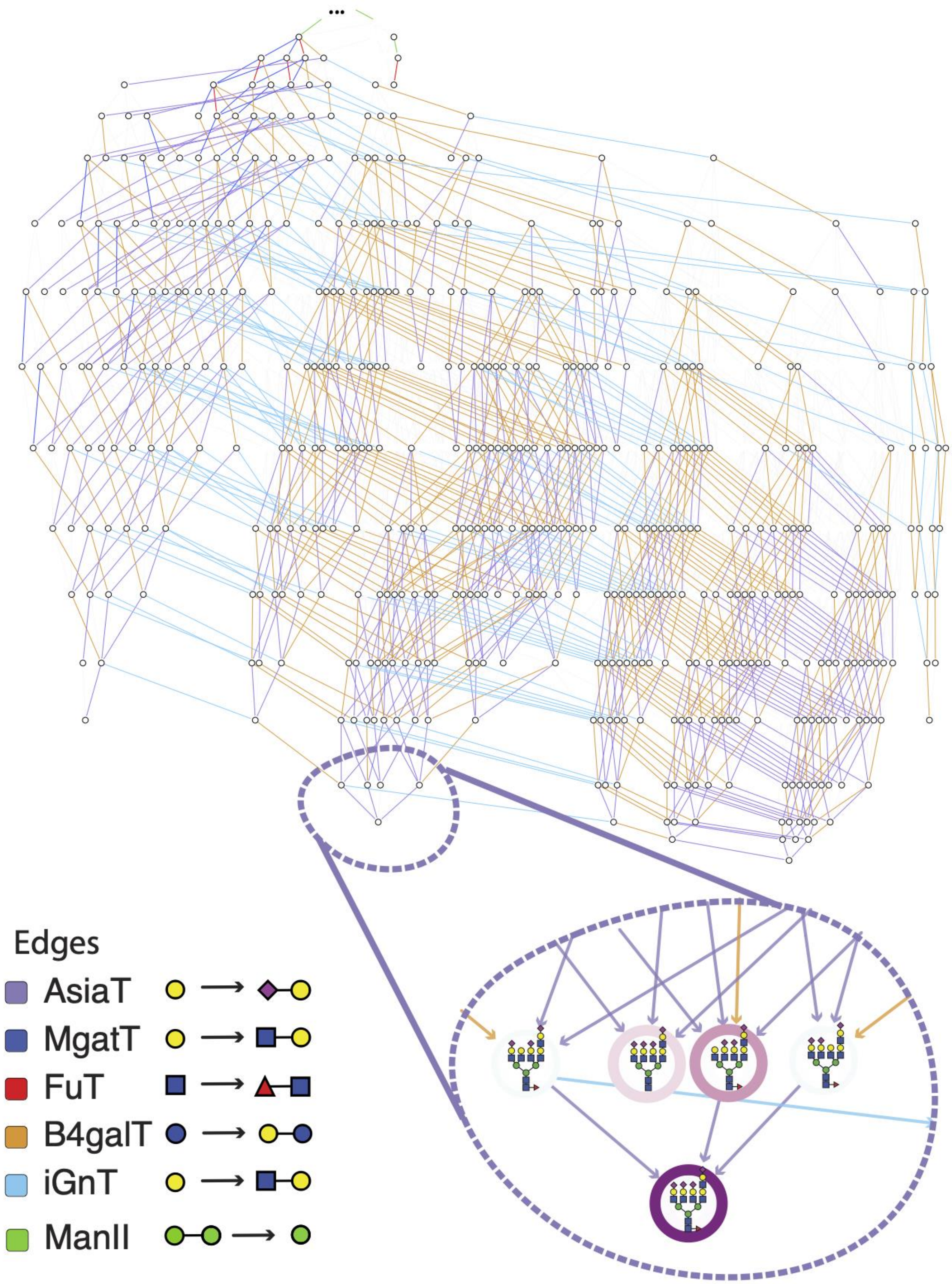
Edges
AsiaT
MgatT
FuT
B4galT
iGnT
ManII

Supplementary Figure 2 | Robustness of glyco-motifs clusters

This is the cluster of glyco-motif vectors for EPO data. The robustness gives the criteria of how many substructure clusters should be generated[1]. The clusters are distinguished if AU (red)=100 (approximately unbiased probability value p<0.01) and then BP (green) (Bootstrap Probability) >15. We get 35 clusters in our EPO data.

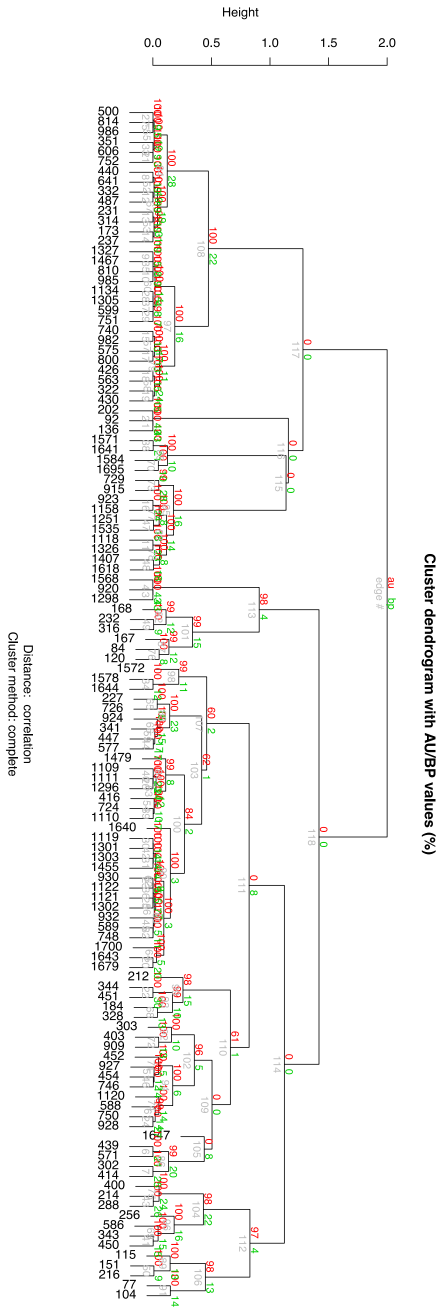

Supplementary Figure 3 | The glycoprofile clustering table with the original glycans.

a-b the clustering of 16 glycoprofiles based on the glycan abundance Pearson correlation and the clustering of glyco-motif profiles based on the motif abundance Pearson correlation. The color represents the correlation from 0 to 1. The colored labels are as same as the colored labels in Fig. 3a. **c** the details of Fig. 3a. The color represents the glycan relative abundance from 0 to 1. The colored labels are as same as the colored labels from Fig. 3a-b. The cluster mainly focuses on the presence/absence of the glycans (clusters 3-10), which means the information of structural similarity tends to be ignored in the clustering. This would drastically limit their analytic power due to the sparsity of comparable consensus glycans. Source data are provided as a Source Data file.

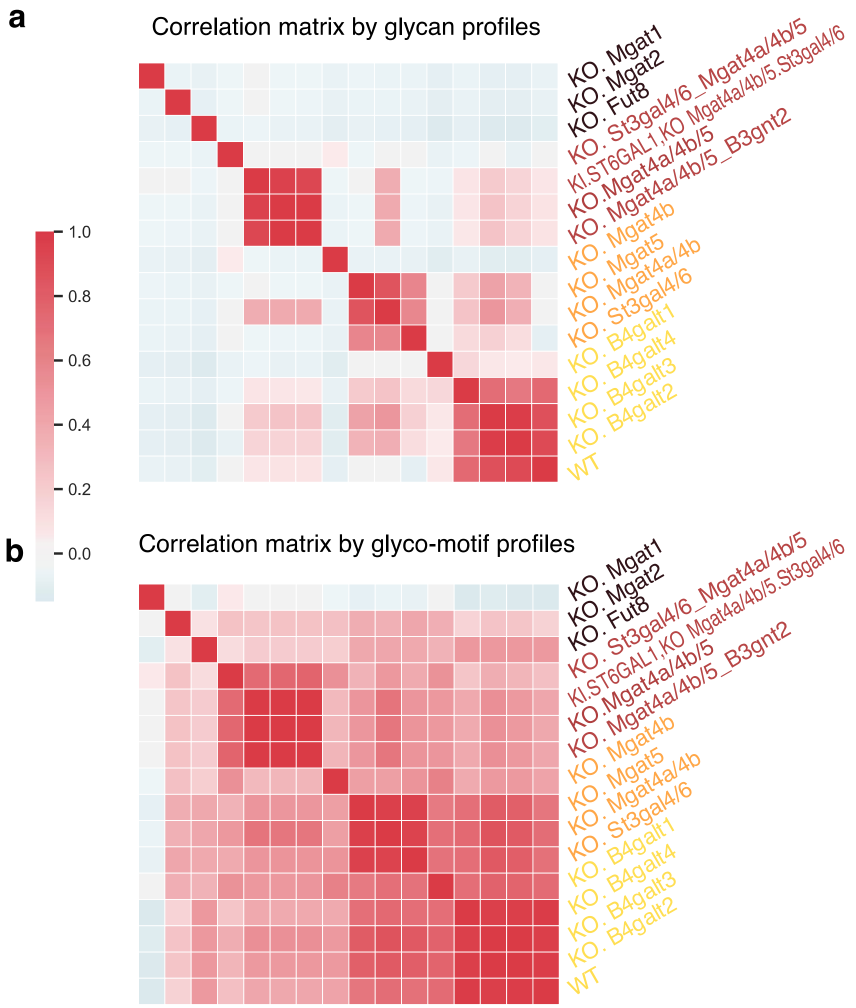

c

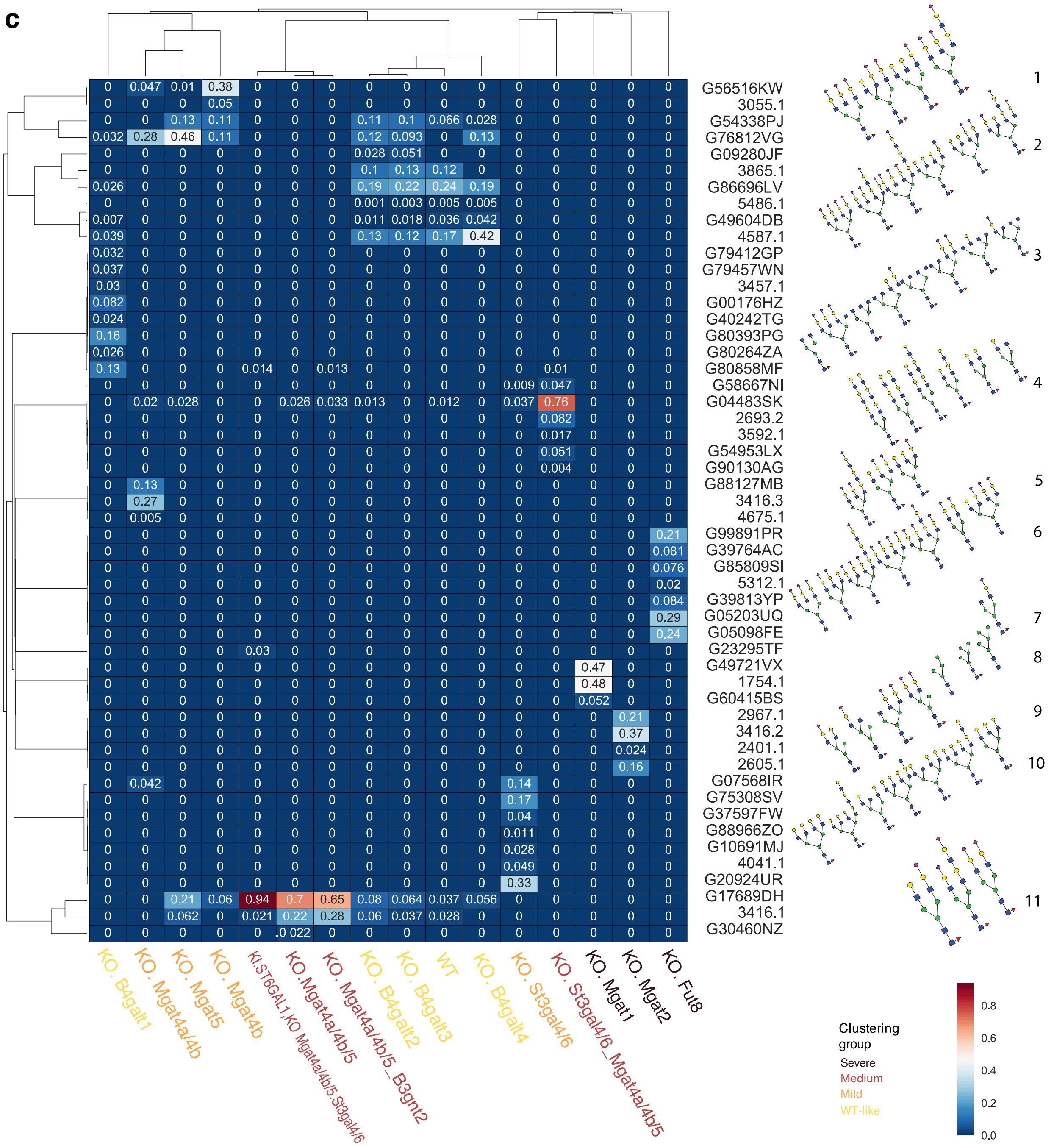
G56516KW
3055.1
G54338PJ
G76812VG
G09280JF
3865.1
G86696LV
5486.1
G49604DB
4587.1
G79412GP
G79457WN
3457.1
G00176HZ
G40242TG
G80393PG
G80264ZA
G80858MF
G58667NI
G04483SK
2693.2
3592.1
G54953LX
G90130AG
G88127MB
3416.3
4675.1
G99891PR
G39764AC
G85809SI
5312.1
G39813YP
G05203UQ
G05098FE
G23295TF
G49721VX
1754.1
G60415BS
2967.1
3416.2
2401.1
2605.1
G07568IR
G75308SV
G37597FW
G88966ZO
G10691MJ
4041.1
G20924UR
G17689DH
3416.1
G30460NZ
1
2
3
4
5
6
7
8
9
10
11
KO. B4galt1
KO. Mgat4a/4b
KO. Mgat5
KO. Mgat4b
KI.ST6GAL1,KO Mgat4a/4b/5.St3gal4/6
KO.Mgat4a/4b/5
KO. Mgat4a/4b/5_B3gnt2
KO. B4galt2
KO. B4galt3
WT
KO. B4galt4
KO. St3gal4/6
KO. St3gal4/6_Mgat4a/4b/5
KO. Mgat1
KO. Mgat2
KO. Fut8
Clustering group
Severe
Medium
Mild
WT-like
0.8
0.6
0.4
0.2
0.0

Supplementary Figure 4 | The clustering robustness.

The robustness is measured with BP (Bootstrap Probability). BP is a measure of the cluster robustness suggesting a significant similarity within clusters and thereby mitigating some challenges of clustering reproducibility. Glyco-motifs abundances showed higher BP than clustering with whole-glycan abundance profiles. In the whole-glycan profile clusters, wild-type (WT) glycoprofiles are closer to the double-knockouts with highly-perturbed glycoprofiles. Double-knockouts are predominantly determined to be strongly perturbed and therefore should not cluster with wild-types in a biologically meaningful clustering. As such, we believe the glycan clustering (which clusters WT with double knockouts) is less interpretable than the glyco-motif clustering which does not include the WT/double-knockout grouping.

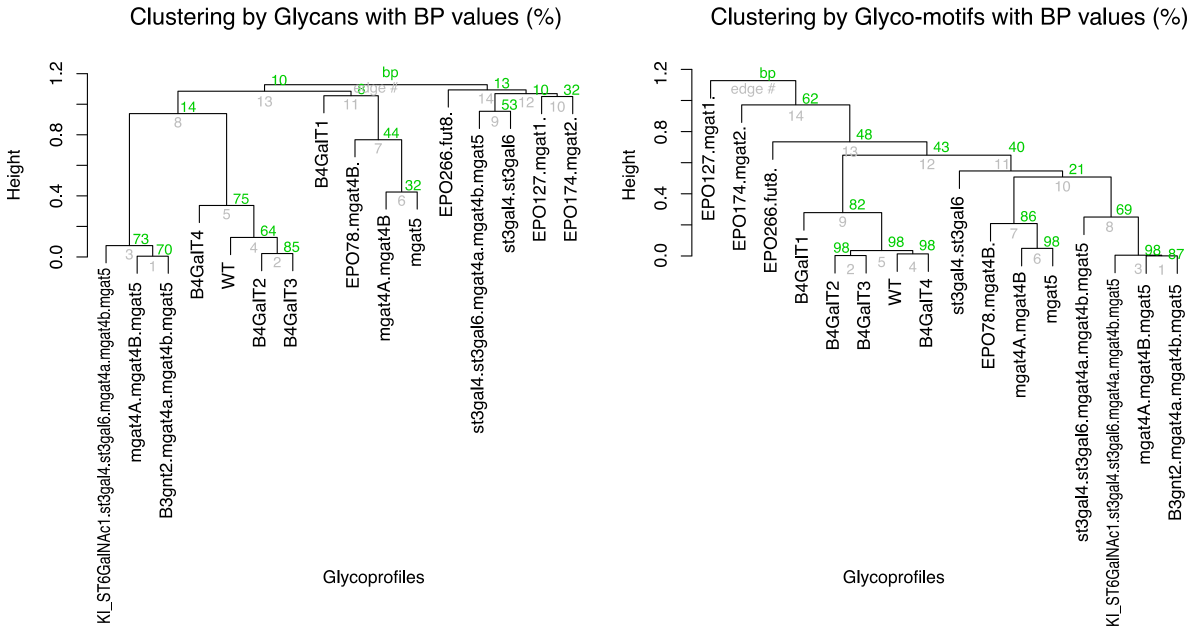

Supplementary Figure 5 | Profile matching between the data from Čaval et al. 2018[2] and GlyCompare

The lightened names are the knockouts that do not have MALDI-TOF data published[2]. The KO name with the same color (for example, in brown, yellow, green, red, purple) are the KO profiles that clustered together. While some glyco-motif clusters can be seen in the glycoprofile clusters, there are important differences, and the glyco-motif clusters provide more information and improved cluster stability. Furthermore, the clustering result based on the glyco-motif was consistent with the clustering based on the native mass spectrometry, except for the Mgat2 knockout and the Fut8 knockout, which considerably changed the glycoprofiles by removing many common glycans. The main reason is that GlyCompare accounts for structural differences caused by each glycosyltransferase. This allows us to evaluate the magnitude of differences between glycans, whether it be between glycans with the same mass but different structural topologies, or subtle structural variations due to single changes in monosaccharides. Therefore, we had a better interpretation of the glycan structure variants across multiple glycoprofiles. All these results demonstrated the excellent performance of our GlyCompare in assessing the structural similarity between different glycoprofiles.

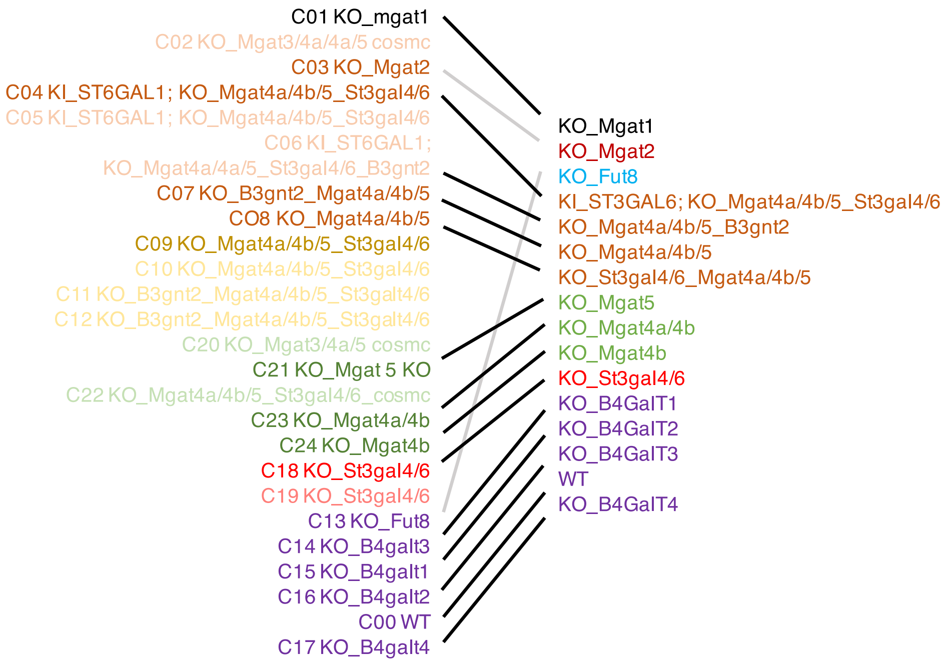
C01 KO_mgat1
C02 KO_Mgat3/4a/4a/5 cosmc
C03 KO_Mgat2
C04 KI_ST6GAL1; KO_Mgat4a/4b/5_St3gal4/6
C05 KI_ST6GAL1; KO_Mgat4a/4b/5_St3gal4/6
C06 KI_ST6GAL1;
KO_Mgat4a/4a/5_St3gal4/6_B3gnt2
C07 KO_B3gnt2_Mgat4a/4b/5
CO8 KO_Mgat4a/4b/5
C09 KO_Mgat4a/4b/5_St3gal4/6
C10 KO_Mgat4a/4b/5_St3gal4/6
C11 KO_B3gnt2_Mgat4a/4b/5_St3galt4/6
C12 KO_B3gnt2_Mgat4a/4b/5_St3galt4/6
C20 KO_Mgat3/4a/5 cosmc
C21 KO_Mgat 5 KO
C22 KO_Mgat4a/4b/5_St3gal4/6_cosmc
C23 KO_Mgat4a/4b
C24 KO_Mgat4b
C18 KO_St3gal4/6
C19 KO_St3gal4/6
C13 KO_Fut8
C14 KO_B4galt3
C15 KO_B4galt1
C16 KO_B4galt2
C00 WT
C17 KO_B4galt4
KO_Mgat1
KO_Mgat2
KO_Fut8
KI_ST3GAL6; KO_Mgat4a/4b/5_St3gal4/6
KO_Mgat4a/4b/5_B3gnt2
KO_Mgat4a/4b/5
KO_St3gal4/6_Mgat4a/4b/5
KO_Mgat5
KO_Mgat4a/4b
KO_Mgat4b
KO_St3gal4/6
KO_B4GalT1
KO_B4GalT2
KO_B4GalT3
WT
KO_B4GalT4

Supplementary Figure 6 | The unscaled cluster abundance related to Fig. 3a

The heatmap of glycan abundance for the thirty-five substructure clusters. The substructures are sorted based on the glycan structure complexity, followed by the number of branches, the degree of galactosylation, sialylation, and fucosylation. While comparing to WT, the weighted average abundance of each cluster is calculated by each column. The color denotes the change of glycan abundance for the comparison of KO vs. WT of the indicated substructure. Source data are provided as a Source Data file.

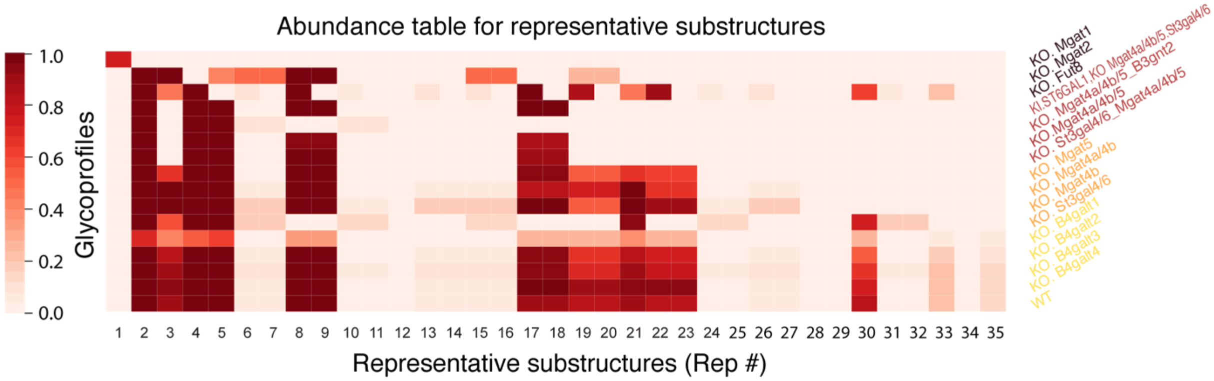

Supplementary Figure 7 | Representative substructure network for KO.Fut8

The differential substructure representative network for the comparison between the Fut8 knockout profile and the WT profile. The z-score rescaled substructure clusters' abundance in Fig. 4a are visualized with a simplified network. The color is defined the same as in Fig. 4a for the fold change of glycan abundance.

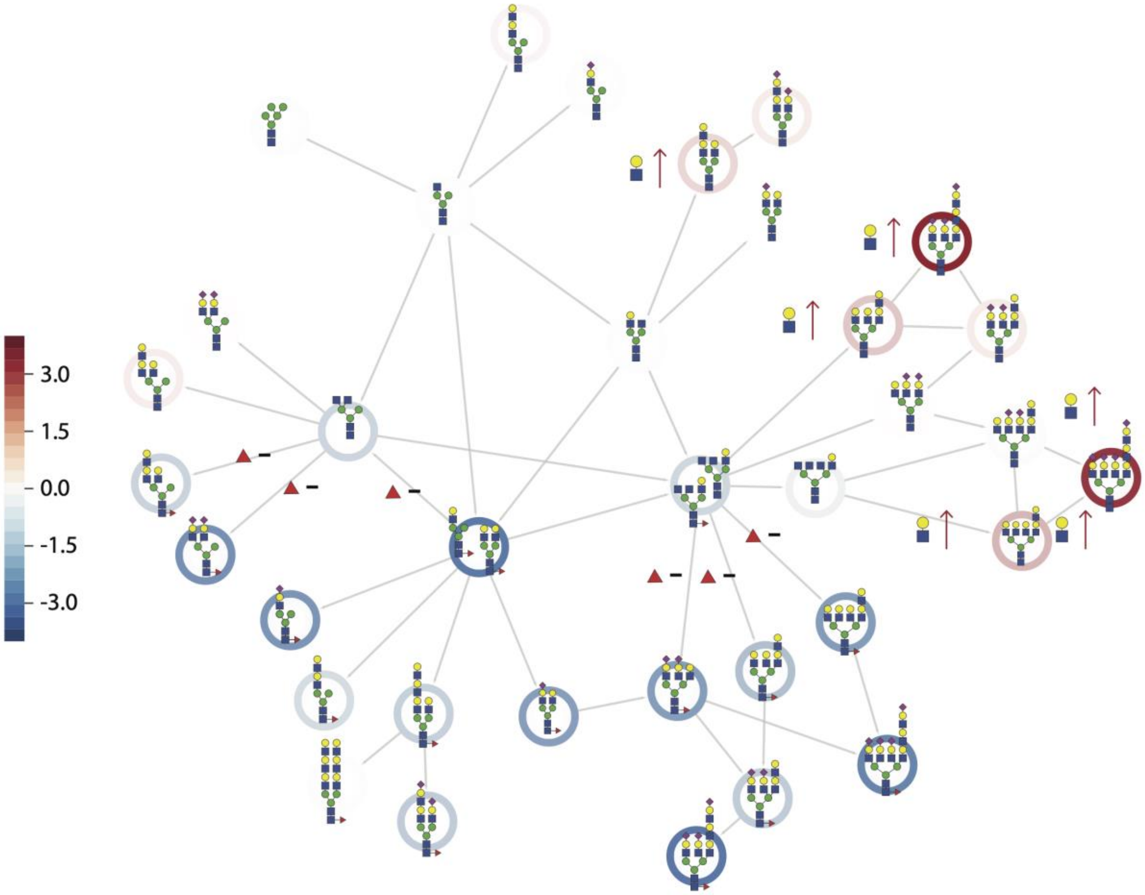
3.0
1.5
0.0
-1.5
-3.0

Supplementary Figure 8 | HMO substructure network with dependent substructure removed

All the glyco-motifs are shown, and redundant nodes are merged. This is a directed-acyclic-graph and the direction goes from top to bottom. An edge with black color is an important edge after merging that indicates the abundance changes. An edge with blue color is an edge that exists before merging that indicates the abundance variation between two substructures.

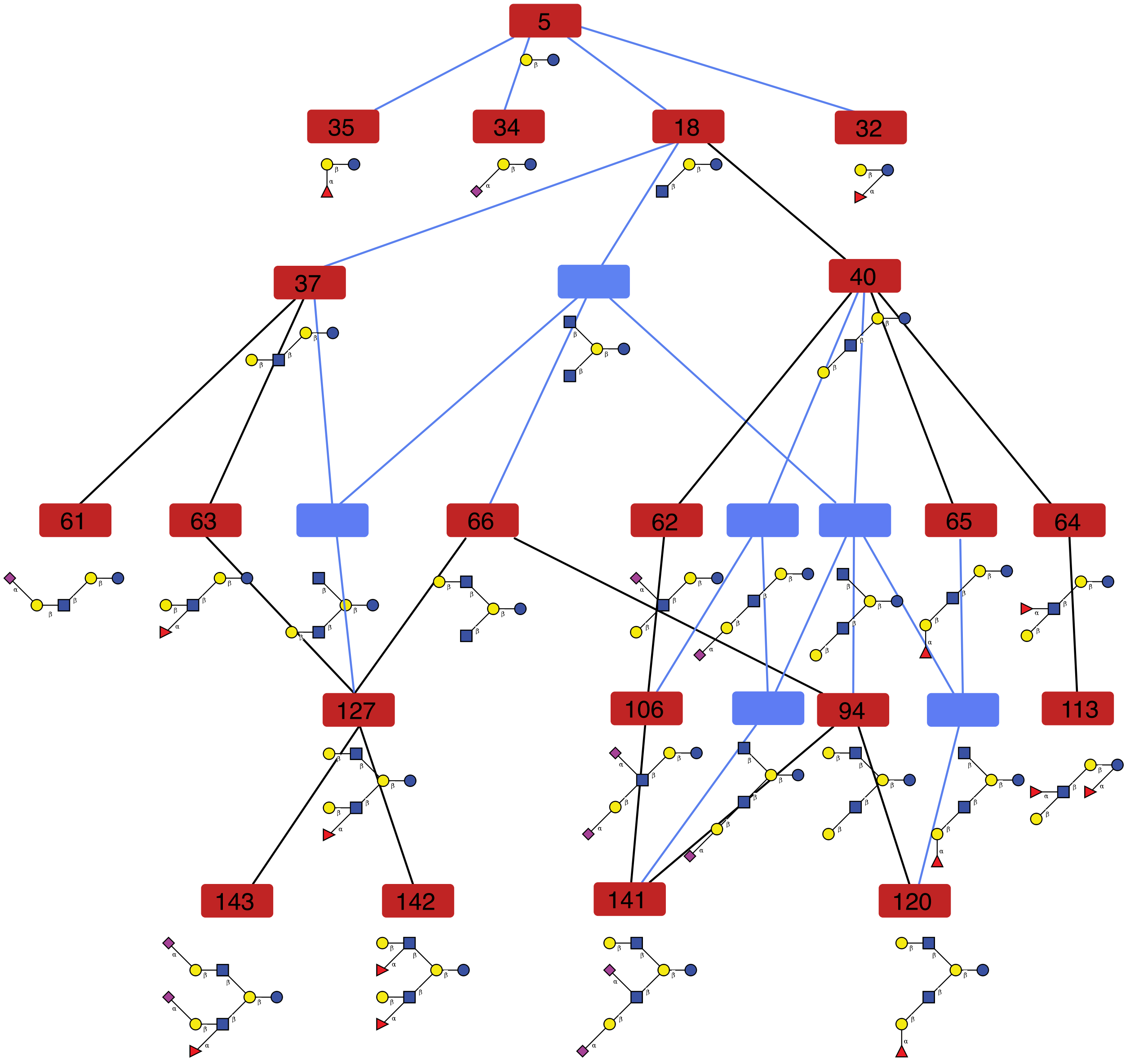

Supplementary Figure 9 | HMO dataset, the clustering of HMO by glycan using Pearson correlation distance

a, c. At the glycan-level, 2-fucosyllactose (2' FL) is the most abundant HMO in secretor mothers while Lacto-N-tetraose (LNT) and LNFPI are the most abundant HMOs in non-secretor mothers. The second major source of variance, DPP, shows a decrease in non-secretor LNFPI. **b, d.** At the substructure level, the clustering recapitulated the results from the raw HMO profiles and the α-1,2 fucosylated substructures were significantly associated with secretor status. The 2'FL substructure (X35) and the LNFPI substructure (X65) are significantly more abundant in secretor milk (Wald p=2.35x10^{-25}, Wald p=5.1x10^{-12} respectively). The substructure abundance successfully reproduces the strongest effects known to be associated with secretor status. Source data are provided as a Source Data file.

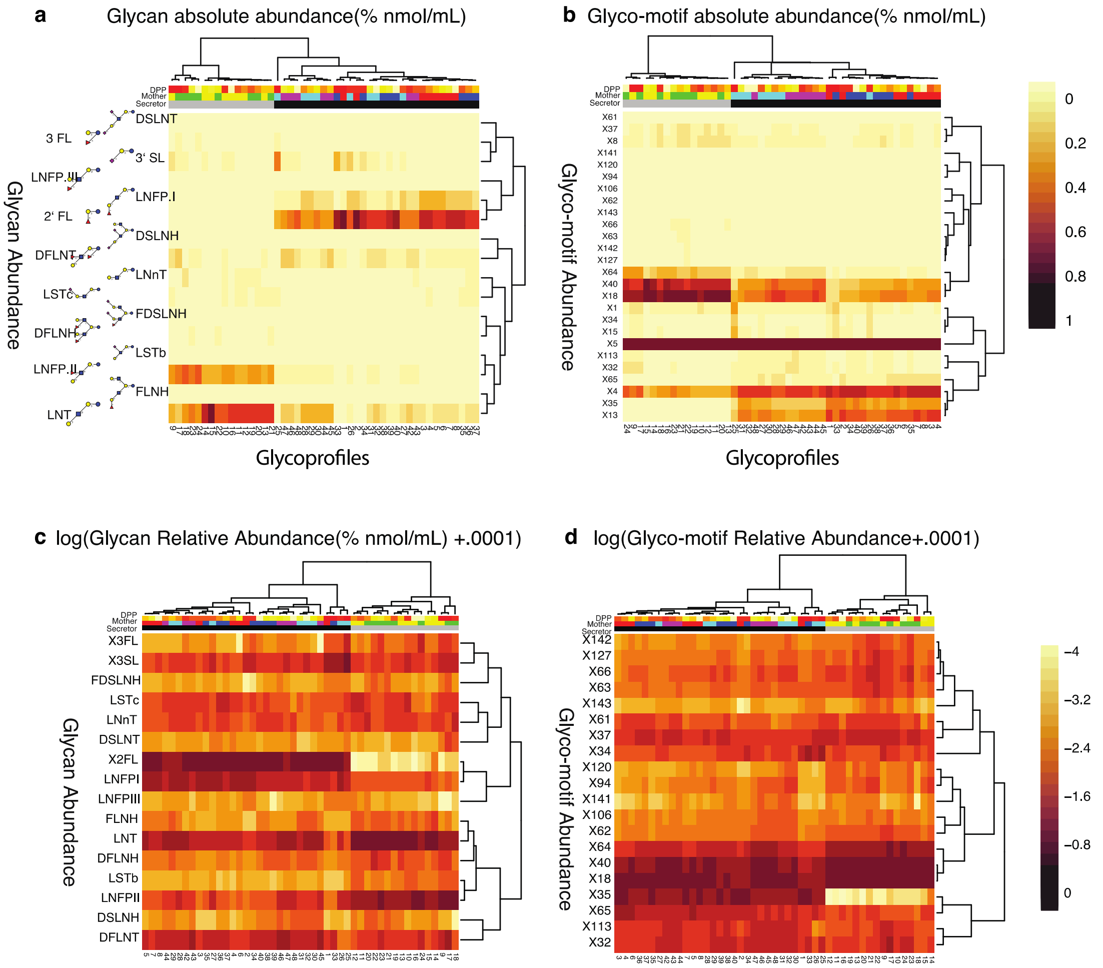

Supplementary Figure 10 | HMO substructure general estimate equation coefficient and p-value plot

Summary of regressions predicting either glycan or motif abundance from Days Postpartum (DPP) and Secretor status. The horizontal axis indicates the coefficient associating either DPP or secretor status with abundance and the vertical axis indicates the significance of that coefficient using the Wald test. Regression models were fitted using Generalized Estimating Equations (GEE) with an exchangeable covariance structure to control for dependency structures within mothers. Colors indicate the identification of the glycan or glycan motif, size indicates the significance of the coefficient and shape indicates if the coefficient was attributed to DPP or secretor status. Models fit to predict glycan abundance (left) were of the form: GEE(z(log(S+ ϵ))) ~ DPP + secretor, while models to predict motif abundance were of the form: GEE(z(log(S+ ϵ))) ~ DPP + secretor. Where, z(x) is a z-score normalization to center and standardize abundance and $\epsilon = 0.001$. This link function was chosen because it fit a normal distribution (Supplemental Table 3a) and allowed for comparisons between regressions. There are some notable consistencies between the motif and glycan level results. As expected, 2'FL and its motif, X35, are both strongly and significantly enriched in secretor status. As are LNFPI and its motif, X65, are also strongly and significantly enriched with secretor status. Conversely, LSTb and X62 are negatively associated with secretor status. DPP has some significant but small negative associations with LSTc, 3'SL, and DSLNT. The 3'SL motif, X34 showed a consistent small negative significant association and the DSLNT motif. Most notably, X1, the sialic acid motif, was strongly negatively associated with DPP suggesting sialylation decreased in these samples over time. Source data are provided as a Source Data file.

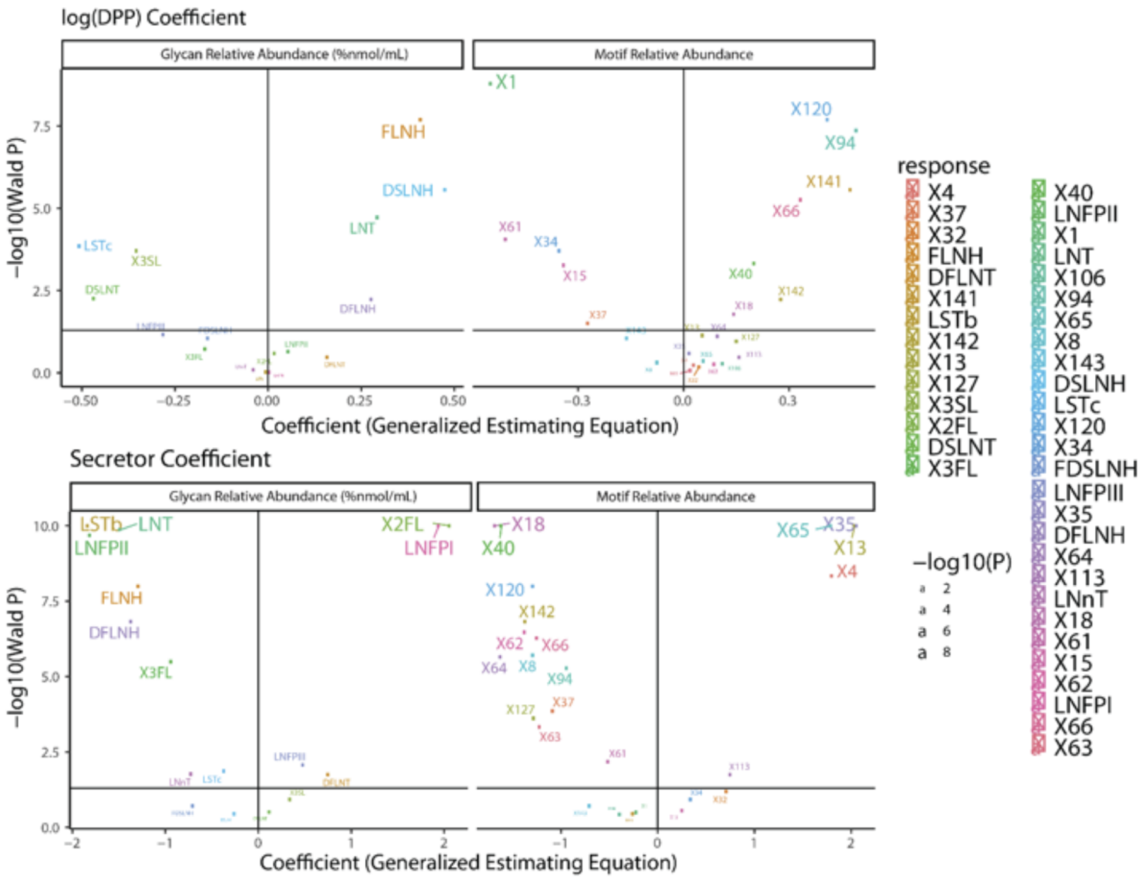
log(DPP) Coefficient
Glycan Relative Abundance (%nmol/mL)
Motif Relative Abundance
−log10(Wald P)
Coefficient (Generalized Estimating Equation)
Secretor Coefficient
Glycan Relative Abundance (%nmol/mL)
Motif Relative Abundance
−log10(Wald P)
Coefficient (Generalized Estimating Equation)
response
X4
X37
X32
FLNH
DFLNT
X141
LSTb
X142
X13
X127
X3SL
X2FL
DSLNT
X3FL
X40
LNFPII
X1
LNT
X106
X94
X65
X8
X143
DSLNH
LSTc
X120
X34
FDSLNH
LNFPIII
X35
DFLNH
X64
X113
LNnT
X18
X61
X15
X62
LNFPI
X66
X63
−log10(P)
2
4
6
8

Supplementary Figure 11 | Partial correlation between known and unknown biosynthesis reactions in N-glycosylation.

Glycan abundance data (MALDI-TOF) from Benedetti et. al. 2017[3] was used to compute partial correlation between glycan abundance (as in the original paper, blue) and glycompare-computed linkage-specified substructure abundance (red). Partial correlations were stratified by prior knowledge, those known and previously characterized were designated the true-positive (T) reactions, while the other uncharacterized reactions were designated false-positive (F). The detailed information about the quartile boundary is provided in the Supplementary Table 4. **a** A panel shows partial correlations that are split by IgG isoforms. The one-sided T.test are performed between the glycan abundance and substructure abundance (IgG1, F p=0.068, T p=0.0039; IgG2, F p=0.27, T p=2.1e-07; IgG4, F p=0.6, T p=4.1e-09;) **b** A panel shows partial correlations split by related glycosyltransferases (B4GALT, T p=1.1e-04; IgG2, T p=1.8e-07). Source data are provided as a Source Data file.

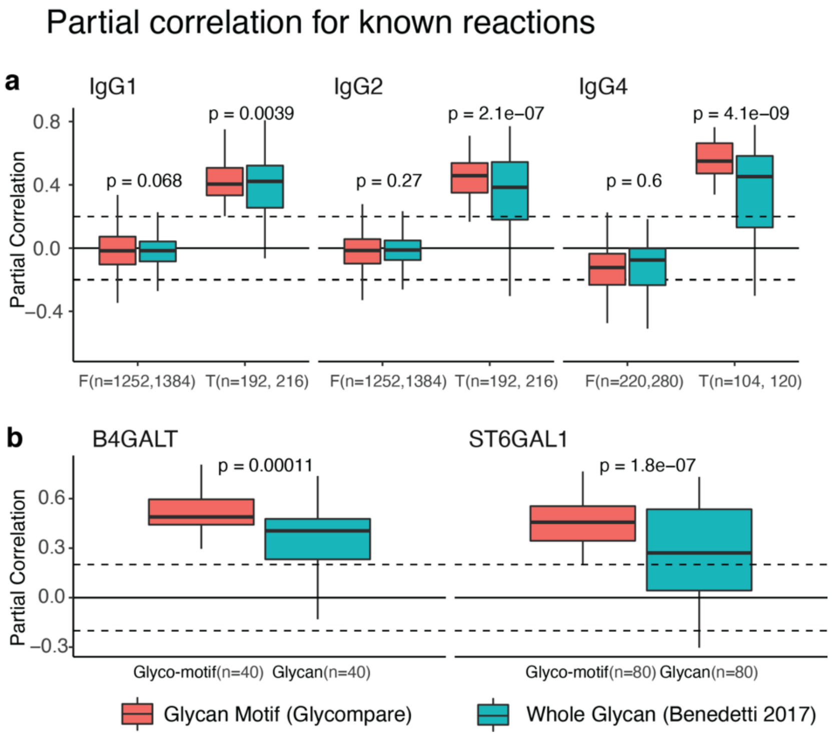

Supplementary Figure 12 | A re-analysis of ganglioside glycolipid abundance pooled across various ceramide types.

Glycolipid and substructure abundance from a lactose root clarified distinct glycosylation in the retina[4]. **a,** Retinal GD3 substructure abundance is enriched in retina across nearly all ceramide species while the same effect is not visible at the whole glycan level. **b,** Retinal GM2 is depleted relative to the proximal ciliary body but the effect is only visible at the substructure level. Ceramide groupings include more than 42 or fewer than 35 Carbons ($C_{>42}$, $C_{<35}$), either 1 or 2 unsaturated bonds (1 unsat., 2 unsat), or groups of specific ceramides with X:Y carbons and unsaturated bonds (e.g. 34:1, (36:1+38:1), or (40:1+40:2). The subjects N=7 for all boxplots. The quartiles information is recorded on the Supplementary Table 5. Source data are provided as a Source Data file.

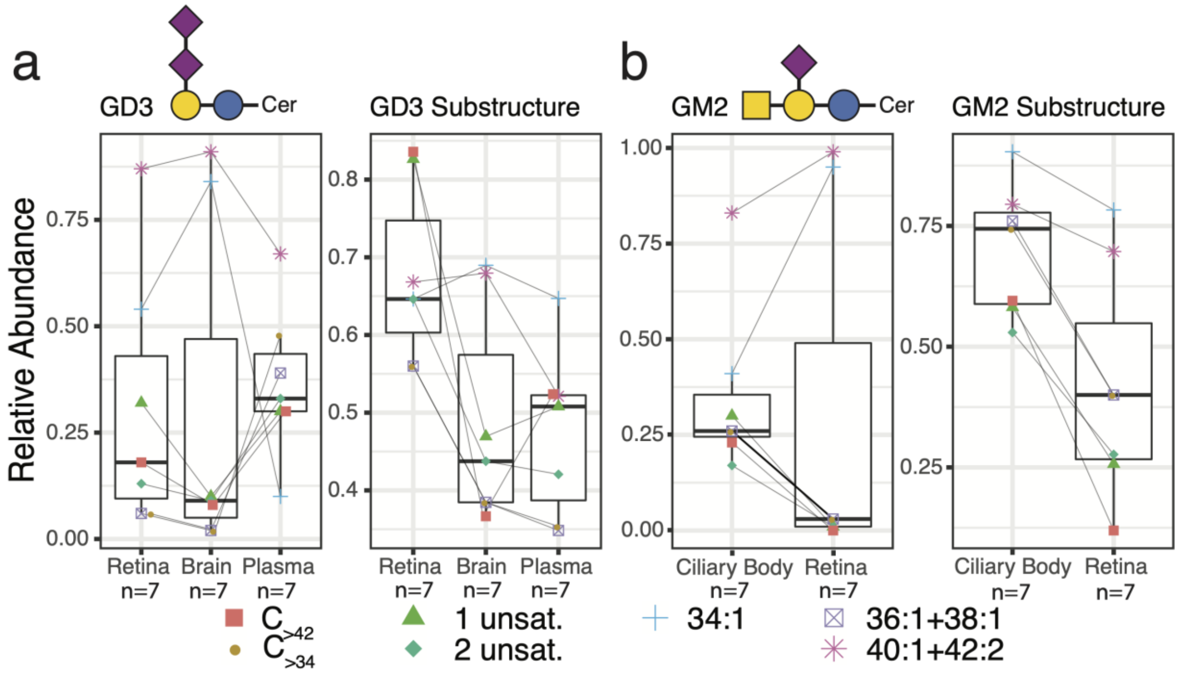

Supplementary Figure 13 | A reanalysis of site-specific N-glycosylation in mouse brain.

a. Compositional site-specific N-glycan data from mouse brain[5]. **b.** The same compositional data was substructure-decomposed to calculate substructure abundances presented in another biclustered heatmap. **c, d.** The Pearson correlation coefficient was calculated for the compositional and composition substructure abundance for each glycosylation site across proteins. The resulting correlation coefficients are presented as biclustered heatmaps. Biclustering used a complete agglomerative approach. See Methods for details.

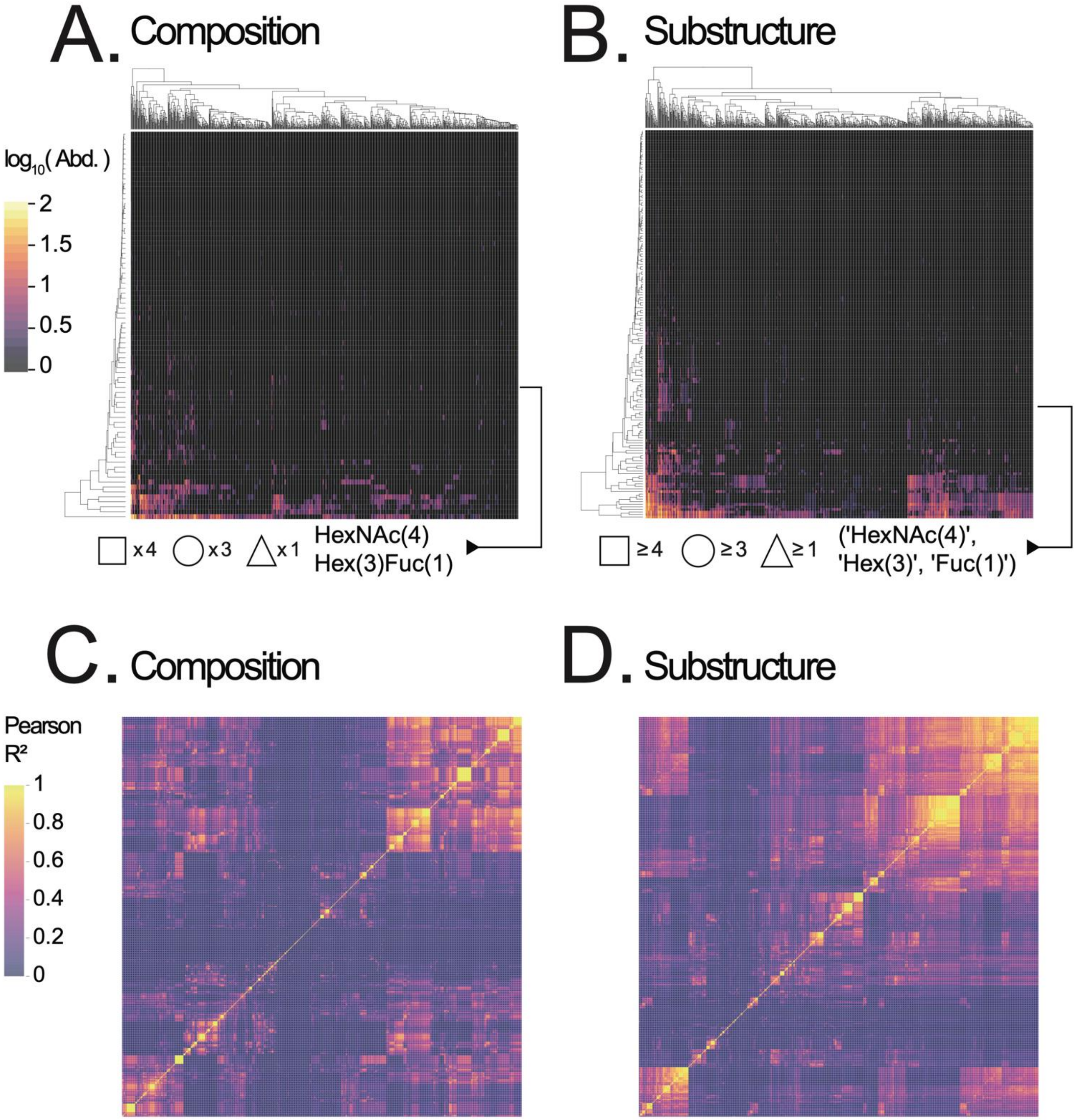
A. Composition
B. Substructure
log10(Abd.)
2
1.5
1
0.5
0
x4
x3
x1
HexNAc(4)
Hex(3)Fuc(1)
≥4
≥3
≥1
('HexNAc(4)',
'Hex(3)', 'Fuc(1)')
C. Composition
D. Substructure
Pearson
R²
1
0.8
0.6
0.4
0.2
0

Supplementary Figure 14 | The workflow of the pipeline. This flowchart provides a basic overview of the glycompare platform. Glycomics data which contain the glycan structure (or compositional) information and abundance information, are fed into the pipeline. The green boxes are the main pipeline function in the glycompare platform. The glycan structures are loaded as glypy.Glycan object at the initialization step. Then, the glyco-motif vectors are generated with the help of the glycan abundance. After that, glyco-motif profiles are delivered to the clustering analysis and statistical analysis modules.

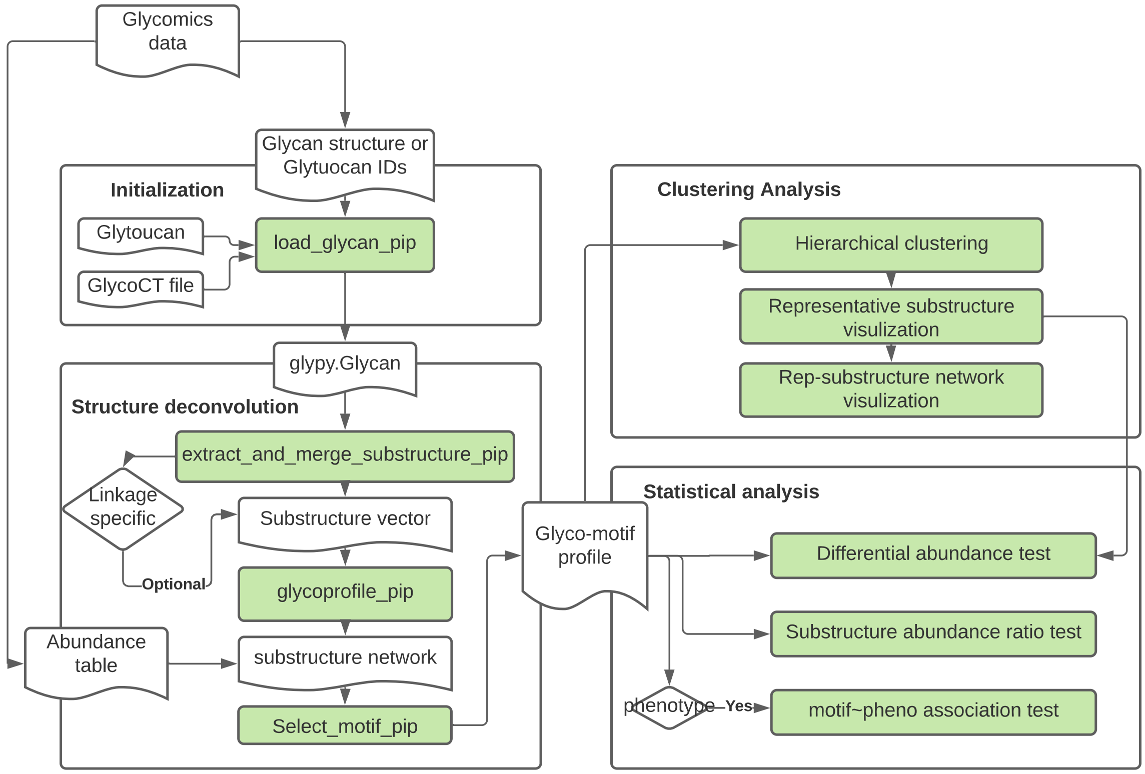

Supplementary Discussion

Re-analysis of ganglioside glycolipid abundance pooled across various ceramide types

When ganglioside and substructure abundance was pooled by ceramide types, we found the GD3 substructure enriched in retina relative to brain and plasma, while the GD3 ganglioside abundance showed no coherent effect (Supplementary Figure 12a). Similarly, the GM2 substructure was enriched across several ceramide types in the Ciliary-body relative to the retina, while the GM2 ganglioside showed no coherent effect (Supplementary Figure 12b). By aggregating over subtypes, we can account for confounding biosynthetic complexity thereby simplifying analyses and making crucial insights more accessible.

Re-analysis of site-specific N-glycosylation in mouse brains

Examining site-specific N-glycan compositional data from rat brain, we found that the decomposition of composition abundance into composition substructure abundance reveals additional potential signal. As previously shown, the sparsity of the abundance matrix decreases, and the comparability of profiles is improved when glycan data is aggregated over substructures (Supplementary Figure 13a, b). Further, the correlation structure of substructure aggregated abundance (Supplementary Figure 13d) appears more robust than its compositional counterpart (Supplementary Figure 13c); there are more clusters with clearer borders, multiple clear off-diagonal clusters and the median R^2 is approximately doubled. While it is possible that the higher correlation is indicative of an increased background, that is unlikely considering the increase in visible correlation is structured, not randomly distributed through the background.

Supplementary References

1. Maechler, M. et al. Cluster: cluster analysis basics and extensions. R package version **1**, 56 (2012).
2. Čaval, T., Tian, W., Yang, Z., Clausen, H. & Heck, A. J. R. Direct quality control of glycoengineered erythropoietin variants. Nat. Commun. 9, 3342 (2018).
3. Benedetti, E. et al. Network inference from glycoproteomics data reveals new reactions in the IgG glycosylation pathway. Nature Communications vol. 8 (2017).
4. Sibille, E. et al. Ganglioside Profiling of the Human Retina: Comparison with Other Ocular Structures, Brain and Plasma Reveals Tissue Specificities. PLoS One 11, e0168794 (2016).
5. Riley, N. M., Hebert, A. S., Westphall, M. S. & Coon, J. J. Capturing site-specific heterogeneity with large-scale N-glycoproteome analysis. Nat. Commun. 10, 1311 (2019).
